# Pathophysiology and the Monitoring Methods for Cardiac Arrest Associated Brain Injury

**DOI:** 10.3390/ijms18010129

**Published:** 2017-01-11

**Authors:** Cesar Reis, Onat Akyol, Camila Araujo, Lei Huang, Budbazar Enkhjargal, Jay Malaguit, Vadim Gospodarev, John H. Zhang

**Affiliations:** 1 Department of Physiology and Pharmacology, Loma Linda University School of Medicine, 11041 Campus Street, Risley Hall, Room 219, Loma Linda, CA 92354, USA; cesarreis@hotmail.com (C.R.); onatakyol@hotmail.com (O.A.); araujo.ct@gmail.com (C.A.); lhuang@llu.edu (L.H.); enkhjargalb@hotmail.com (B.E.); jay.malaguit@gmail.com (J.M.); vgospodarev@llu.edu (V.G.); 2 Department of Anesthesiology, Loma Linda University Medical Center, Loma Linda, CA 92354, USA; 3 Department of Neurosurgery, Loma Linda University School of Medicine, Loma Linda, CA 92354, USA

**Keywords:** ICP monitoring, cerebral autoregulation, Electrophysiologic monitoring, brain injury after cardiac arrest, brain oxygen monitoring, microdialysis, metabolic tracing and cardiac arrest brain injury, intracranial pressure

## Abstract

Cardiac arrest (CA) is a well-known cause of global brain ischemia. After CA and subsequent loss of consciousness, oxygen tension starts to decline and leads to a series of cellular changes that will lead to cellular death, if not reversed immediately, with brain edema as a result. The electroencephalographic activity starts to change as well. Although increased intracranial pressure (ICP) is not a direct result of cardiac arrest, it can still occur due to hypoxic-ischemic encephalopathy induced changes in brain tissue, and is a measure of brain edema after CA and ischemic brain injury. In this review, we will discuss the pathophysiology of brain edema after CA, some available techniques, and methods to monitor brain oxygen, electroencephalography (EEG), ICP (intracranial pressure), and microdialysis on its measurement of cerebral metabolism and its usefulness both in clinical practice and possible basic science research in development. With this review, we hope to gain knowledge of the more personalized information about patient status and specifics of their brain injury, and thus facilitating the physicians’ decision making in terms of which treatments to pursue.

## 1. Introduction

Cardiac arrest (CA) has a yearly incidence of approximately 50–110 per 100,000 people worldwide [1] and has become a leading cause of coma and admission to the intensive care unit (ICU). Progress in advanced life support, access to emergent coronary angiography, and support for cerebral perfusion have contributed to an increase in CA survival [2]. Due to the severity of brain dysfunction after CA from global ischemia-reperfusion injury, there is a need to understand the prognosis of patients in the ICU and their chances of survival. Caregivers want to ascertain a patient’s chance of survival and how serious their condition is [3]. Thus, integration of tracing methods such as electroencephalography (EEG), Intracranial pressure monitoring (ICP), brain oxygen monitoring, and microdialysis may provide valuable information regarding brain dysfunction after CA. In this review, we will provide an updated critical overview of the available tracing methods for brain injury after CA in adults.

Explosive growth of technology and information has occurred over the years and clinicians approach their daily practice differently compared to previous generations of medical practice. A reasonable explanation takes into consideration the fact that advances in pre-mortem non-invasive diagnostic methods are available for doctors, with less need to perform pre-mortem biopsies. Advances in imaging, clinical laboratory studies, and surgical and nonsurgical tissue sampling techniques over the past several decades provide the tools needed to better diagnose and detect pre mortem pathologies, thus yielding a greater number of ante mortem diagnoses and shifting physicians’ attitude away from needing to perform a detailed autopsy to acquire information [4]. In addition, doctors are not getting trained in autopsies since the Accreditation Council for Medical Education (ACGME) program has eliminated the autopsy requirement in residency education [5]. Nonetheless, some data support the continued value of autopsy in spite of medical diagnostic advancements because they serve to increase the accuracy of death certificates, enhance identification of emerging or reemerging pathogens/disease, and provide an educational format for clinicians, pathologists, and students [5]. Another challenge is the large heterogeneity in the populations of humans ending up with cardiac arrest, which makes choosing a representative experimental model of cardiac arrest difficult. Results are profoundly heterogeneous, since there is an array of comorbidities. There is a need to improve experimental models since animal models are designed mainly to serve a monothematic purpose. This could be done by combining common comorbidities such as diabetes mellitus, obesity, and hypertension in an experimental animal model to better represent the heterogeneity of the human population experiencing cardiac arrest [6].

The PubMed search for this review focused on original articles, review papers and clinical trials published in peer-reviewed journals; however, there are several challenges in identifying the exact intracranial events after cardiac arrest. Major problems regarding this issue include inadequate pre-mortem tissue sampling, difficulties in brain tissue acquisition and analysis after cardiac arrest, challenges in establishing a representative in vivo experimental model that directly projects and describes either molecular or gross events following cardiac arrest, and the existence of population heterogeneity in post cardiac arrest clinical research. In order to minimize and exclude these effects in this review, we primarily focused on stroke-models like global hypoxia-ischemia and oxygen deprived cell line studies. In this review, we also involved and cited the clinical trials whose targeted population has a diagnosis of cardiac arrest with various etiologies.

## 2. Methods

For this review, multiple searches were conducted in order to find the appropriate articles for each subsection. In the section entitled Pathophysiology, a PubMed Boolean search was performed using “cardiac arrest and pathophysiology and brain”, 1820 articles were found. Another Boolean search was performed using “global brain ischemia and pathophysiology”, and 1852 articles were found. Forty-three sources were used. In the section entitled Electrophysiologic Monitoring of Brain Injury after Cardiac Arrest, PubMed search criteria included “EEG and cardiac arrest”. These keywords were used to find review papers and original articles in last ten years and 437 articles were found, and 19 papers were selected. In the section entitled Intracranial Pressure Monitoring and Cerebral Autoregulation, PubMed research database was searched using the following keywords: “Cerebral edema and cardiac arrest” and found 382 results. Review papers were avoided, and this narrowed down the search to 3 papers of interest based on criteria of clinical study and use of experimental animal models. “Intracranial pressure monitoring” search found 4872 results. Review papers were avoided, and this narrowed down the search to 10 papers based on criteria of clinical studies and experimental animal models. “Cerebral autoregulation and cardiac arrest” search found 210 results. Review papers were avoided, and this narrowed down the search to one paper based on criteria of clinical study. The total number of citations used for “Intracranial Pressure Monitoring and Cerebral Autoregulation” was 14. In the section entitled Brain Oxygen Monitoring, several searches were performed. A search using keywords “Near-infrared spectroscopy and Cerebral Oximetry” yielded 700 results, and 4 were selected. A search using keywords “Cerebral ischemia and oximetry” found 340 articles, and 3 were selected. A search using keywords “mean arterial pressure and cardiac arrest” found 1478 articles, and 2 were selected. Lastly, a search using keywords “cerebral oximetry and cardiac arrest” yielded 121 results and 6 were selected. Categories for inclusion included clinically relevant and translatable, including animal studies, monitoring methods already being implemented in the clinical setting, measured or was involved in continuous measurement of brain tissue oxygen content/saturation, and more recent publications took priority. In the section entitled Metabolic Imaging Modalities for Cardiac Arrest Brain Injury, PubMed search used the following keywords, “metabolic imaging and cardiac arrest and brain” and 44 search results were found. An additional set of keywords included “molecular imaging and cardiac arrest and brain” and 16 search results were found. Total number of citations used for this section was 6. In the section entitled Microdialysis, a PubMed Boolean search was conducted using the keywords “Microdialysis and cardiac arrest”. Eighty-five articles were found. Excluding papers except for those published in last five years left 70 articles. Inclusion criteria also included using reviews for background information and then focusing on clinical trials. Fourteen papers were utilized in this section.

## 3. Pathophysiology

**Figure 1:**
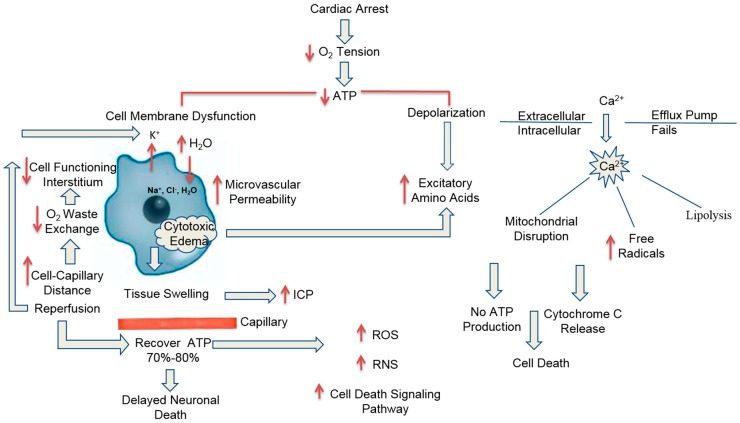
Demonstrates the cascade of effects after cardiac arrest and decreased oxygen tension has on the brain cellular function due to decreased ATP (Adenosine triphosphate), increased excitatory amino acids, and the failure of Calcium efflux pump to work. ICP (intracerebral Pressure), ROS (reactive oxygen species), RNS (reactive nitrogen species). The thin arrows indicate the increase or decrease of either a substance or diagrams the consequences of cardiac arrest.

The brain is extremely susceptible to global cerebral ischemia associated with cardiac arrest. Hosmann and colleagues, working with monkeys, found that consciousness was lost within 10 s after the onset of circulatory arrest and EEG activity becomes isoelectric within 20 s [7]. There was a rapidly declining cerebral tissue oxygen tension to 0 in about 2 min in a pig model of cardiac arrest [8]. When undergoing completed global cerebral ischemia, energy substrates adenosine triphosphate (ATP) in canine brain tissues depleted rapidly to 25%–30% of baseline level within the first 4 min (Figure 1) [9,10]. Once the storage of ATP is completely consumed, the dysfunction of cell membrane ion pumps and the disruption of cellular ionic homoeostasis occur (Figure 1). The interstitial K^+^ concentration rapidly increases, membrane depolarization and a large influx of Na^+^, Cl^−^ and water into cells, constituting cytotoxic edema [11,12]. The calcium efflux pumps fail and voltage-gated calcium channels open, contributing to a massive cytosol Ca^2+^ accumulation followed by the release of intracellular excitatory amino acids such as glutamate and aspirate by presynaptic terminals [13]. When activated by glutamate, *N*-methyl-d-aspartate (NMDA) receptors activate Ca^2+^ ion channels and further increase Ca^2+^ conductance into the intracellular space; α amino-3-hydroxy-5-methyl-4-isoxasolepropionic acid (AMPA) receptors activate Na^+^ ion channels allowing sodium influx. As a critical factor, elevated levels of intracellular Ca^2+^ exert cytotoxic effects at multiple levels, leading to the mitochondrial dysfunction and free radical production [14], lipolysis and production of free fatty acids (FFAs) associated with phospholipases (PLA2) activation [15]. Mitochondrial Ca^2+^ overload cause the induction of inner mitochondrial membrane permeability transition (MPT), which then leads to disruption of mitochondrial membrane integrity, irreversible oxidative damage, and the loss of ATP production, finally resulting in cell death (Figure 1) [16,17]. If reperfusion starts, it enhances the production of a large amount of reactive oxygen species (ROS) originating primarily from mitochondrial complexes I and III of the electron transport chain, in the forms of a superoxide anion (O_2_^−^), H_2_O_2_, and hydroxyl radical (OH^−^) [18]. It overwhelms the endogenous mitochondrial and cytoplasmic scavenging systems causing oxidative damage to the mitochondria and consequently the cell [19]. Other highly reactive free radicals, namely reactive nitrogen species (RNS) are produced by protein nitrosylation due to the reaction of nitric oxide (NO) and superoxide anions, which can also lead to the dysregulation of cellular homeostasis. The major targets of ROS/RNS are lipids, and the peroxidative action of ROS promotes the inactivation of key metabolic enzymes that regulate glucose metabolism. ROS/RNS can also cause cellular damage and death in cerebral ischemia and reperfusion indirectly by activating a variety of signaling pathways [20,21].

The initial cell swelling (cytotoxic edema) is a translocation of interstitial water into intracellular compartment following the disturbance of ionic hemostasis (Figure 1); only after the beginning of reperfusion a net accumulation of brain water interstitially occurs following increased microvascular permeability [22]. Increased cerebral microvascular permeability has been observed in the experimental CA models [22,23,24]. Consistently, in CA patients, computed tomography (CT) and magnetic resonance imaging (MRI) scanning have also shown diffuse brain edema formation predicting poor neurological outcomes [25,26,27]. Brain edema can lead to an elevated ICP due to the limited space for expansion in the crania [27,28]. Tissue swelling increases capillary–tissue diffusion distance and impairs oxygen (O_2_)/waste exchange, eventually leading to loss of tissue function [22]. The mechanisms of CA-induced cerebral edema formation are likely multifactorial. Recently, perivascular pool of aquaporin-4 (AQP4) has been suggested as one key mechanism because this brain predominant water channel controls the rate limit of water influx during cerebral edema formation and it is the regulating site of osmotic agents for brain water efflux [29].

If global ischemia endures without resuscitation, severe cerebral ischemia renders the mitochondria completely dysfunctional for ATP production, leading to necrotic cell death [30]. Although reperfusion upon cardiopulmonary resuscitation (CPR) and return of spontaneous circulation (ROSC) can recover neuronal ATP levels to at least 70%–80% of normal levels (Figure 1), it does not completely restore cell function as delayed neuronal death follows in the vulnerable brain regions [20].

An important feature of global ischemia reperfusion injury after CA and resuscitation is a substantial delay between the end of short insult and cell death. This “delayed apoptotic neuronal death” generally occurs several days and is most evident in the CA1 region of the hippocampus. It also occurs within the parts of the CA3 region of hippocampus, striatum and layers 2 and 5 in cerebral cortex [31,32]. The disruption of mitochondrial membrane integrity underlies the main mechanism of neuronal apoptosis by releasing pro-apoptotic proteins such as cytochrome C and apoptosis-inducing factor Smac [33,34]. Cytochrome C interacts with the cell death protein (CED-4) homolog, Apaf-1, and deoxyadenosine triphosphate, forming the apoptosome and leading to caspase-9 activation [35,36,37,38]. Caspase-9 then activates caspase-3, followed by downstream caspase-2, -6, -8, and -10 activations [39]. Smac binds inhibitor-of-apoptosis proteins (IAPs), thereby promoting activation of caspase-3 [40,41]. The downstream caspases can cleave many substrate proteins including poly (ADP-ribose) polymerase (PARP) [42], leading to DNA injury and subsequently apoptotic cell death. Emerging evidence also implicates the intrinsic caspase independent mechanism underlying neuron apoptosis. The apoptosis inducing factor (AIF), endonuclease G and Bcl-2/adenovirus E1B 19 kDa-interacting protein (BNIP3) constitutes this set of proapoptotic proteins released from mitochondrial transition pores [18]. In addition, apoptotic signaling can be trigged extrinsically through members of the death receptor family, such as Fas, and tumor necrosis factor (TNF) receptor. The binding of extracellular Fas ligand (FasL) to Fas-associated death domain (FADD) protein can activate procaspase-8 and subsequent caspase-3 pathway. Caspase-8 is also able to truncate and activate Bcl-2 interacting domain (BID), a pro-apoptotic member of Bcl-2 family, subsequently initiating cytochrome C release and apoptosis [18].

**Figure 2:**
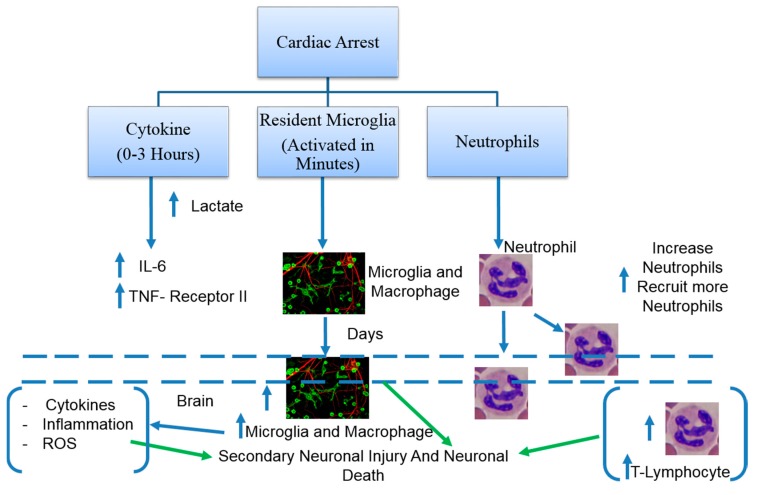
Cardiac arrest and the resulting activation of the inflammatory cascade is demonstrated on the figure above. Inflammation is depicted as a factor that contributes to secondary neuronal injury and death. The dashed line represents a division of the brain and periphery. This figure shows the migration of cells from the blood into the brain. The arrows in green demonstrate the factors that will lead to Secondary Neuronal Injury and Neuronal Death. Penetration of Microglia in the brain also leads to stimulation of Cytokines, inflammation, and ROS (reactive oxygen species). The upward facing arrows indicate the increase in a certain factor or cell after cardiac arrest. Gerry Shaw, Microglia and neurons, 25 July 2005 by creative commons; Hematologist, Segmented Neutrophils, 31 August 2009, Creative Commons.

Inflammation plays an important role in global brain ischemia. The post-resuscitation diseases have been suggested as a sepsis-like syndrome, involving a systemic inflammatory response in a cardiac arrest patient [43]. After CA and resuscitation, a rapid elevation of various cytokines and soluble receptors occurs in the bloodstream as early as 3 h (Figure 2). The levels of cytokines such as interleukin-6 and soluble tumor necrosis factor receptor II are well correlated with tissue hypoxia marker lactate [43]. In a mice model of cardiac arrest, resuscitation resulted in the rapid infiltration of neutrophil and pro-inflammatory T-lymphocytes into the brain, starting within 3 h of resuscitation and lasting for the three days [44,45]. The activation of resident microglia initiated within minutes after the onset of cardiac arrest and developed over hours to days after resuscitation (Figure 2) [46]. The activation of Toll-like receptors and subsequently nuclear factor-κB contribute to the pro-inflammatory genes transcription in human cell lines [47]. Such Toll-like receptors/NF-κB signaling on monocytes contributes to post-resuscitation syndrome [48]. Infiltrating peripheral leukocytes and brain residual microglia and macrophages further trigger infiltration of peripheral immune cells, by releasing ROS and pro-inflammatory cytokines, which in turn affect microglial activation. This vicious cycle contributes to ongoing secondary neuronal injury [49,50].

In the next sections, we will review the tracing methods that monitor brain injury and edema, facilitating the understanding of some of the pathophysiological mechanism above. In addition, we will discuss the methods available to monitor brain function after CA and their application and updates in research and clinical practice.

## 4. Electrophysiologic Monitoring of Brain Injury after Cardiac Arrest

EEG, an electrical phenomenon of the brain, was discovered more than one century ago, and has lasted several decades. Scientists are trying to use EEG as a prognostic predictor and find out its advantages after CA and human brain injury. Currently, the most useful method of Electrophysiological Monitoring of Brain Injury after CA is an EEG. Most patients succumb due to hypoxic brain damage; even after successful cardiopulmonary resuscitation following CA, prediction of brain injury during or after CA remains an important issue guiding the decision to continue or withdrawal treatment, and organ donation. The prognostic prediction of EEG is an important diagnostic criterion of brain injury, such as clinical symptoms and biochemical markers. Technical advantages of EEG recording including continuous EEG, quantitative EEG, and amplitude-integrated EEG are used to predict patient outcomes after CA. Moreover, somatosensory, motor, auditory, and visual evoked potentials in EEG provide information about degree of functional damage of brain after CA [51].

Human and animal studies have shown that EEG is a well-described method to monitor acute brain injury after CA. The studies demonstrated that EEG is a useful monitor for seizure and ischemia detection and, has a well-described role for EEG in convulsive status epilepticus after CA [52]. Recently, several review papers have summarized Electrophysiologic Monitoring of Brain Injury after CA with therapeutic hypothermia (TH), a standard method for treatment of CA [51,52]. Thus, most EEG records have been done in CA with therapeutic hypothermia in humans. Recent studies showed that bispectral index (<40, a calculated summary of raw EEG data including frequency and amplitude) [53,54], burst suppression (a quantitative EGG parameter) [55,56], periodic discharges (commonly recognized patterns of abnormal EEG) [57,58], epileptiform discharges (an uncommon EEG pattern including periodic spike, sharp and slow wave complex and more common in people with epilepsy) [59], stimulus-induced rhythmic periodic, or ictal discharges (a spectrum of stimulation-induced EEG pattern) [60] in the EEG records after successful resuscitation with non-therapeutic hypothermia may indicate poor prognosis. In addition, a non-reactive EEG [61,62] may predict poor prognosis in patients without therapeutic hypothermia, while rhythmic delta activity may indicate better prognosis [55,58] after CA [63].

The absence or discontinuation of reactivity in continuous EEG is a strong predictor of outcome independent of temperature and sedatives [64]. Bilateral absence of cortical N20 component, an early cortical response in EEG, on median nerve somatosensory evoked potentials (SSEPs) was one of the most specific predictors of poor outcome in the 2006 American Academy of Neurology practice parameters [65]. However, one study found that bilateral absence of N20 potentials does not guarantee poor outcome amongst therapeutic hypothermia-treated CA patients [66]. Recently, a case report in a young patient showed that highly-preserved anterior cortico-thalamic integrity is associated with the presence of conscious awareness independent from the degree of injury to other brain areas [67]. Borjigin and colleagues described the CA stages, which began at the last heartbeat in the EEG, and showed that gamma oscillations indicate a highly aroused brain. Furthermore, they demonstrated that the mammalian brain has the potential for high levels of internal information processing during clinical death [68].

Cellular electrophysiology, such as multi-unit activity and local field potentials, has strong potential for improving prognostication after CA, but they still require further investigation before being translated into in vivo studies and clinical practice [51]. Studies showed that a continuous EEG is more precise for evaluation of brain injury after CA [69], especially to detect non-convulsive epileptic status. This status is a critical condition that could be induced by CA, causing exacerbation of brain injury, and poor outcomes [70]. Diagnosis and anti-convulsive epileptic status therapy might be one best way to protect brain injury after CA.

More comparable studies are recommended for scientific evidence to accurately describe EEG characterization after CA with and without therapeutic hypothermia. They also need to describe outcomes after CA that are altered under hypothermic conditions. Several limitations should be considered in the further studies. As a diagnostic tool, waveform-based EEG analysis is subjective and laborious, with results depending on the interpreter’s expertise [71]. Most studies published are case reports. There is a lack of large number studies, and animal experiments in various clinical context using different measures of the brain activity and function collected. In addition, data are heterogenetic by the use of various devices [71]. The optimal montage and number of electrodes to record EEG in the ICU is uncertain and EEG recording is dependent on a certain number of electrodes, their nature, form and placement. Thus, perspective, direction of clinical application of EEG, and studies should be considered in order to establish standardized settings with constant time of EEG recording after CA, and use of continued EEG recordings as a prognostic predictor in EEG, such as N20 and cerebral recovery index. Future improvement of EEG using current advantages of EEG devices might be finest and specific prediction method of Electrophysiological Monitoring of Brain Injury after CA.

## 5. Intracranial Pressure Monitoring and Cerebral Autoregulation

Cerebral edema is an important cause of secondary brain injury following CA and must be carefully monitored in order to decrease the risk of coma and chance of mortality amongst individuals that experience such a devastating event [27,29].

Increased intracerebral pressure (ICP) is not a direct result of cardiac arrest, as evidenced by a clinical study conducted by Sakabe and colleagues who found that ICP persistently remained below 20 mmHg in five out of six patients following cardiopulmonary resuscitation [72]. Nonetheless, cerebral edema can still occur and therefore an increase in ICP, due to hypoxic-ischemic encephalopathy induced changes in brain tissue. Brain swelling was found in a group of patients that experienced CA as a result of respiratory distress based on results of a clinical study conducted by Morimoto and colleagues, which could eventually lead to cerebral herniation and brain death [27]. ICP monitoring is an important clinical tool due to a strong association between high ICPs (>20 mmHg) and increased rates of mortality based on outcomes in patients with severe traumatic brain injury [73]. Indications for ICP monitoring, especially in post-CA patients presenting with initially unremarkable cranial computed tomography (CT) neuroimaging, can be determined based on a predictive model, which includes patients’ age (>40 years), systolic blood pressure (<90 mmHg) and signs of decerebrate or decorticate posturing [74,75].

The most commonly used ICP monitoring devices are categorized based on their location of placement: parenchymal and ventricular. Both types are minimally associated with infection and bleeding with greater risk of complications typically seen in use of extra cranial ventricular drain ICP monitors [76]. In order to decrease the chance of causing further complications in patients that are already in critical condition due to intracranial hypertension, non-invasive ICP monitoring techniques are currently being developed. These include transcranial Doppler devices, optic nerve sheath diameter (ONSD) measurements and even pupillometry [77,78,79]. Unfortunately, due to limited studies and lack of reproducible results, these novel non-invasive ICP monitoring devices are not widely used in clinical practice.

Besides the obvious danger of cerebral herniation, the other concern associated with having increased ICP is a decrease in cerebral perfusion pressure (normal = 60 mmHg), which is derived from mean arterial pressure (MAP)—ICP. In a non-pathologic state, cerebral autoregulation is responsible for maintenance of cerebral blood flow (CBF) in light of the constantly fluctuating MAP. While conducting a clinical study, Sundgreen and colleagues found that in a majority of patients in the acute phase of cardiac arrest, cerebral autoregulation was either absent or right-shifted, thereby suggesting that MAP should be maintained at higher levels to maintain proper cerebral perfusion, which was measured via transcranial Doppler ultrasonography [80].

However, the aforementioned advances in ICP monitoring and understanding of cerebral autoregulation would have never seen the light of day unless results from animal studies were translated into clinical trials, interventions and innovations.

Experimental studies are often a required precursor to clinical trials that test differences in intervention therapies’ effectiveness in a clinical setting. Mirski and colleagues questioned the difference in effectiveness between administration of mannitol and hypertonic saline in reduction and control of ICP, the latter of which was found to be more effective in rats, at least in the first 8 h [81]. While other rodent studies conducted by Hiploylee and colleagues came to the conclusion that there are no differences when comparing ICP readings from epidural vs. intraparenchymal monitoring locations [82], thereby giving clinicians a basis to confirm such findings through clinical trials, yielding results that would allow less invasive ICP monitoring options for patients requiring such care. Furthermore, studies which utilized ultrasound in ICP monitoring and a novel method of epidural ICP recording, such as those conducted by Limbrick, and Uldall, respectively, are the driving force behind progress and innovation when it comes to novel methods for long-term ICP monitoring, which is highly sought after in both the basic science, as well as in the clinical setting [83,84].

## 6. Brain Oxygen Monitoring

While methods exist for monitoring cerebral oxygen content perioperatively, no standardized method of continuous cerebral oxygen monitoring currently exists for patients undergoing an out-of-hospital (OHCA) or in-hospital CA (IHCA). Parnia and colleagues state that the ability to detect and quantify cerebral ischemia in real-time, during CPR is of vital clinical importance [85]. Patients, with a post-clinical determination of CA have suboptimal levels of cerebral oxygen content which may lead to secondary ischemic injuries (i.e., sterile inflammatory cascade).

Many of the more established methods of continuous cerebral oxygen monitoring are invasive, such as monitoring catheters, and or unfeasible (e.g., constant fMRI, CT monitoring) for both out-of-hospital and in-hospital CA patients. In addition, neuromonitoring such as transcranial Doppler sonography and jugular oxygen content (SjvO_2_) are unavailable to out-of-hospital patients [86]. In addition, jugular oxygen content (SjvO_2_) is unavailable to those experiencing OHCA [86]. For instance, the treatment of brain injuries such as traumatic brain injury allows for the placement of monitoring catheters such as the Neurovent-PTO and Licox [87]. Despite the accuracy of regional oxygen saturation provided by probes such as these, it might not be clinically prudent for patients concurrently undergoing both OHCA and/or IHCA. In other words, the accuracy of these oxymetric probes is hindered by their invasiveness and inability to be utilized during CPR.

A non-invasive method of accessing cerebral oxygen content may come from determining mean arterial pressure (MAP). Kilgannon and colleagues postulated whether an association between mean arterial pressure (MAP) and neurological outcome could be found [88]. In the observation of CA patients over a period of 6 h post-return-of-spontaneous-circulation (ROSC), they discovered that 40% of patients with an average MAP pressure above 70 mmHg were associated with a good neurological outcome [88]. A counter to the conclusion by Kilgannon and colleagues argues that central venous pressure (CVP) is a more accurate indicator of cerebral oxygen saturation than MAP [89]. A pilot study conducted by Ameloot and colleagues found that a prolonged CVP over 5 mmHg increased the likelihood of recovery with a poor neurological outcome [89]. Data analysis of simultaneously recorded CVP and cerebral tissue oxygen (SctO_2_) content via near-infrared spectroscopy (NIRS) showed an association between a high CVP (>20 mmHg) and a decrease in SctO_2_ content [89]. Additional analysis between CVP and MAP showed CVP to associate more closely to changes in SctO_2_ [89]. Unfortunately, the measurement of CVP requires the insertion of a venous catheter, a procedure that cannot be conducted on OHCA patients, specifically during CPR. However, multifactorial measurements of SctO_2_ with respect to MAP and CVP may be of an increased benefit to IHCA patients.

Studies have emerged regarding the feasibility and accuracy of near-infrared spectroscopy (NIRS) for non-invasive cerebral oximetry [90]. NIRS utilizes infrared light (700–1100 nm) to determine the difference in absorption between oxyhemoglobin and deoxyhemoglobin [91]. The use of near-infrared spectroscopy during CPR has produced multiple studies resulting in a correlation between higher regional oxygen saturation and a higher chance of return of spontaneous circulation [85,92,93]. However, another study indicated that the high regional oxygen saturation and possibility of return of spontaneous circulation was not significant to determine survivability to discharge [94].

There are pitfalls with cerebral oximetry with near-infrared spectroscopy. Meng and colleagues [95] suggests that the regional area sampled by near-infrared spectroscopy is superficial and may not detect deeper regions that may be vulnerable to ischemic insults post-CA. However, more studies involving near-infrared spectroscopy both during CPR and post-return of spontaneous circulation with larger sample sizes should be conducted to determine whether a high regional oxygen saturation increases a likelihood of a favorable neurological outcome.

## 7. Metabolic Imaging Modalities for Cardiac Arrest Brain Injury

Potential prediction of neurologic outcome after CA induced brain injury has been one of the main aims of advanced neuroimaging techniques. Evidence from clinical studies has pointed out the first 72 h after cardiopulmonary resuscitation to be a poor prediction for cerebral prognosis. Thus, reducing the lack of certainty in neurologic prognosis prediction should be the leading purpose of advanced molecular imaging techniques where maintenance and modulation of cellular pathways causing brain injury can improve neurologic outcomes as well [96].

Brain injury following CA involves periods of global hypoxia-ischemia, reperfusion recovery, glutamate consumption, and GABA-induced pathways [97]. Yi Zhang and colleagues compared ventricular fibrillation and asphyxial CA brain injury models in pigs with 18F-fluorodeoxyglucose PET/CT technique to evaluate the metabolic activity in the residual cerebral tissue. The metabolic activity in two different CA models was found decreased compared to sham in the parietal and frontal lobe as well as brain stem and cerebellum at 24 h after spontaneous recirculation. An additional reduced cerebral glucose metabolism in the asphyxial CA group had been detected to be more significant than in the ventricular fibrillation group. On the other hand, authors emphasized the longer CPR duration in the asphyxial CA group compared to the ventricular fibrillation group and, therefore, its association with poor neurologic prognosis [98]. However, an older and very limited sample sized clinical study by Schaafsma and colleagues reported no beneficial effects of 18F-fluorodeoxyglucose PET technique in means of specifying neurologic prognosis after CA [99].

Clinically, David KH and colleagues reported a broad literature review in order to correlate the association between levels of evidence and quality of previous studies for predicting the accurate neurologic outcomes with molecular neuroimaging techniques in post-CA patients. This review discussed the pros and cons of Positron Emission Tomography (PET) and Single-Photon Emission Computed Tomography (SPECT) in prognostication. However, limited sample sizes, variability in imaging times, and inadequate standardization methods in previous clinical research were present [100]. Krep and colleagues investigated the combination of diffusion-weighted imaging (DWI), ^1^H-proton spectroscopy (H-MRS) and perfusion-weighted imaging (PWI) in an experimental cat study and monitored the variations of regional cerebral blood flow after cerebral recirculation along with lactate and creatinine measurements during the first 6 h after CA. Despite the favorable results, the lower statistical power and inadequate model standardization through the experiment are the main restricted factors for their results [101].

Magnetic resonance spectroscopy (MRS) is a favorable advanced neuroimaging technique for assessing metabolite concentration alterations in ischemic brain injury. In means of CA brain injury, Tang and colleagues [102] analyzed brain metabolites after CA and exhibited lower levels of NAA/Cr (reduced *N*-acetyl aspartate (NAA)-to-creatine ratio) and NAA/Cho (reduced *N*-acetyl aspartate (NAA)-to-Cho ratio) at 6 h after spontaneous recirculation although Cho/Cr levels was found increased. Furthermore, in that study, hypothermia has a lessening effect on NAA/Cr decrease and Cho/Cr increase after CA. Parallel to their study, Su and colleagues used single voxel proton magnetic resonance spectroscopy in CA pig model and found lactic acid production was reduced in hypothermia group [103]. Chan K and colleagues designed an experimental rat study aimed to investigate the H-MRS efficacy for evaluating neuroprotective metabolic changes during hypothermia with CA. Authors demonstrated the transient changes of glucose, choline, tau, myo-inositol, NAA and lactate in cerebral cortex and thalamus as a result of hypothermia [104]. More animal and clinical research with magnetic resonance spectroscopy are necessary in order to establish as a gold standard for monitoring the therapeutic effects of hypothermia and enhance the metabolic impacts of hypothermia during CA brain injury [102].

## 8. Microdialysis

Microdialysis is a method of neuromonitoring consisting of a probe covered in a semi-permeable membrane, which is inserted into the interstitial tissue of the brain. A solution, usually normal saline or artificial cerebral spinal fluid, is perfused through the probe and into the tissue, allowing cerebral fluid, along with some small molecules, to passively diffuse into the membrane where the extracellular concentration of solute is measured. Common solutes of interest include free ions, neurotransmitters, and other biomarkers and metabolites [105].

**Figure 3:**
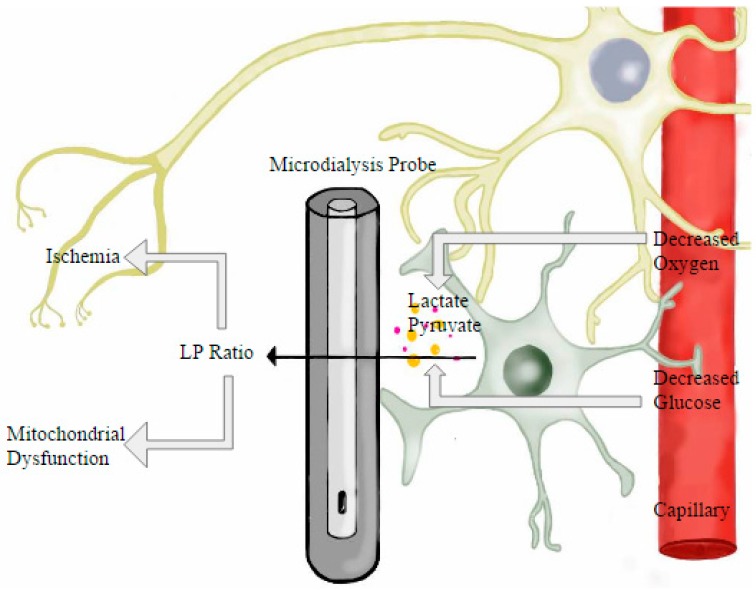
Figure demonstrating the result of decreased brain oxygen and consequent lactate production. A microdialysis probe detects biomarkers, such as Lactate/Pyruvate ratio (LP) that will indicate ischemia or mitochondrial dysfunction.

CA often leads to a cessation of blood flow to the brain, creating areas of ischemia and consequently brain damage. Reperfusion is typically the first in the line of treatments administered; yet it is not without risk. Reperfusion injury includes a broad description of damages that can occur with restoration of cerebral blood flow. Although these molecular events are largely unknown, research in this field is quickly growing. During ischemia, cellular function is altered and one of the known pathways of damage is that of mitochondrial dysfunction. The downstream cascade is believed to begin in the mitochondria, the lack of oxygen results in anaerobic respiration, and in time, cell death [106]. When treating acute brain injury it is imperative to manage these downstream cascades, such as inflammation and edema, in order to prevent the worsening of the initial condition. Through closely observing the physiological changes occurring in the injured brain, it becomes possible for physicians to determine the patients’ exact needs and develop an effective treatment plan that may prevent or lessen the effects of secondary injuries [107]. The most common application of microdialysis is thus measuring cerebral metabolism through observing the levels of lactate (Figure 3), glycerol, pyruvate, glutamate [108], even free ions in the extracellular space [109], and glucose [110].

Although it has been used extensively as a research tool, further developments of microdialysis allow it to be used in clinical settings to better monitor patients who have suffered severe brain injury. Changes in levels of lactate, pyruvate, glycerol and glutamate act as biomarkers and allow clinicians to determine the current state of a patient’s brain, as well to possibly aid in the intervention of secondary injury such as hypoxia, glucose depravation [111] edema, and inflammation [107]. By monitoring the biochemical state of the brain, it is possible to further examine the mechanisms in action, and provide more targeted treatment. A common measurement used to monitor cerebral metabolism is the ratio of lactate to pyruvate, or LPR (Lactate Pyruvate ratio). An increase in this ratio is correlated with neuronal decline due to ischemia or post-ischemic mitochondrial dysfunction [112]. If the ratio is increased due to an increase in lactate, while maintaining a relatively normal level of pyruvate, it indicates increased mitochondrial dysfunction (Figure 3); however if there is an increase in ratio with a normal lactate concentration and a marked decrease in pyruvate, ischemia is the outcome indicated [113]. Two Danish studies, one by Nielsen and the other by Jacobsen supported the bedside use of microdialysis to distinguish between metabolic changes relating to ischemia and mitochondrial dysfunction [112,114].

Hosmann and colleagues used rodents to create a microdialysis CA model, using microdialysis as a neuro-monitor to determine cerebral metabolism during baseline, CA, cardiopulmonary resuscitation, reperfusion, and death. Significant results include the possible distinction between survivors and non-survivors. Non-survivors demonstrated with a marked increase in glutamate compared to surviving rodents within 8 min of CPR. On the other hand, survivors were characterized with higher cerebral and peripheral lactate and glucose concentrations. According to the authors, the usefulness of microdialysis outweighed its invasiveness, as it is able to detail the metabolic time course, as well giving unique insight into chemical changes in the brain [115].

A Viennese pilot trial used microdialysis to examine metabolic changes during ventricular fibrillation CA (VF-CA), to compare two different treatments (extracorporeal life support (ECLS) and cardiopulmonary resuscitation (CPR)), as well as to monitor resuscitation and the return of spontaneous circulation. There were no significant differences between the extracorporeal life support and cardiopulmonary resuscitation treatment groups; all metabolic changes during each step were consistent between the groups. Glucose, glutamate and lactate-pyruvate ratios were the biomarkers used in this study. During CA, glucose decreased dramatically, and after resuscitation, increased back to normal levels. LPR had a five-fold increase from baseline levels, and peaked 8 min after return of spontaneous circulation. Glutamate also had a marked increase (3.5-fold) after CA [116].

Overall, microdialysis has proven useful in clinical settings to monitor cerebral metabolism in critically ill neurological and neurosurgical patients, enabling physicians to provide more effective treatment depending on which biochemical signals are present [117]. However, recent studies have demonstrated the growing applications for this tool, used not only for detection of ischemia and distinguishing ischemia from mitochondrial dysfunction, but also detection of successful reperfusion, even comparing different methods of resuscitation [116]. The growing trend of extended neuromonitoring including devices such as microdialysis is used to gain more personalized information about the patient and their specific brain injury, and thus facilitating the physicians’ decision making in terms of which treatments to pursue [118]. The addition of microdialysis to other neuromonitors aids physicians in creating individualized treatments bedside, which remains a promising step in bettering patient outcomes [119].

## 9. Conclusions

With the challenges faced by the limitations that each method has, there is a strong need to stimulate research towards brain monitoring after CA that can generate enough robust data to support the use of brain monitoring and be able to predict in hospital and long term prognosis in patients affected by brain injury after CA [3]. Notwithstanding the above methodological limitations, reliance on a multimodal approach allows incorporation of relevant information at the present moment to maximize brain monitoring after CA. In this review, we have focused on describing the pathophysiologic mechanisms associated with brain edema after CA and the tracing methods such as brain oxygen monitoring, EEG, and ICP that are needed to measure and evaluate the consequences of CA in the brain. Even though there is a strong need for more methods to accurately give information about injury after CA, the methods presented above are, currently, the key resources used in ICU around the world.
